# Ensembl 2021

**DOI:** 10.1093/nar/gkaa942

**Published:** 2020-11-02

**Authors:** Kevin L Howe, Premanand Achuthan, James Allen, Jamie Allen, Jorge Alvarez-Jarreta, M Ridwan Amode, Irina M Armean, Andrey G Azov, Ruth Bennett, Jyothish Bhai, Konstantinos Billis, Sanjay Boddu, Mehrnaz Charkhchi, Carla Cummins, Luca Da Rin Fioretto, Claire Davidson, Kamalkumar Dodiya, Bilal El Houdaigui, Reham Fatima, Astrid Gall, Carlos Garcia Giron, Tiago Grego, Cristina Guijarro-Clarke, Leanne Haggerty, Anmol Hemrom, Thibaut Hourlier, Osagie G Izuogu, Thomas Juettemann, Vinay Kaikala, Mike Kay, Ilias Lavidas, Tuan Le, Diana Lemos, Jose Gonzalez Martinez, José Carlos Marugán, Thomas Maurel, Aoife C McMahon, Shamika Mohanan, Benjamin Moore, Matthieu Muffato, Denye N Oheh, Dimitrios Paraschas, Anne Parker, Andrew Parton, Irina Prosovetskaia, Manoj P Sakthivel, Ahamed I Abdul Salam, Bianca M Schmitt, Helen Schuilenburg, Dan Sheppard, Emily Steed, Michal Szpak, Marek Szuba, Kieron Taylor, Anja Thormann, Glen Threadgold, Brandon Walts, Andrea Winterbottom, Marc Chakiachvili, Ameya Chaubal, Nishadi De Silva, Bethany Flint, Adam Frankish, Sarah E Hunt, Garth R IIsley, Nick Langridge, Jane E Loveland, Fergal J Martin, Jonathan M Mudge, Joanella Morales, Emily Perry, Magali Ruffier, John Tate, David Thybert, Stephen J Trevanion, Fiona Cunningham, Andrew D Yates, Daniel R Zerbino, Paul Flicek

**Affiliations:** European Molecular Biology Laboratory, European Bioinformatics Institute, Wellcome Genome Campus, Hinxton, Cambridge CB10 1SD, UK; European Molecular Biology Laboratory, European Bioinformatics Institute, Wellcome Genome Campus, Hinxton, Cambridge CB10 1SD, UK; European Molecular Biology Laboratory, European Bioinformatics Institute, Wellcome Genome Campus, Hinxton, Cambridge CB10 1SD, UK; European Molecular Biology Laboratory, European Bioinformatics Institute, Wellcome Genome Campus, Hinxton, Cambridge CB10 1SD, UK; European Molecular Biology Laboratory, European Bioinformatics Institute, Wellcome Genome Campus, Hinxton, Cambridge CB10 1SD, UK; European Molecular Biology Laboratory, European Bioinformatics Institute, Wellcome Genome Campus, Hinxton, Cambridge CB10 1SD, UK; European Molecular Biology Laboratory, European Bioinformatics Institute, Wellcome Genome Campus, Hinxton, Cambridge CB10 1SD, UK; European Molecular Biology Laboratory, European Bioinformatics Institute, Wellcome Genome Campus, Hinxton, Cambridge CB10 1SD, UK; European Molecular Biology Laboratory, European Bioinformatics Institute, Wellcome Genome Campus, Hinxton, Cambridge CB10 1SD, UK; European Molecular Biology Laboratory, European Bioinformatics Institute, Wellcome Genome Campus, Hinxton, Cambridge CB10 1SD, UK; European Molecular Biology Laboratory, European Bioinformatics Institute, Wellcome Genome Campus, Hinxton, Cambridge CB10 1SD, UK; European Molecular Biology Laboratory, European Bioinformatics Institute, Wellcome Genome Campus, Hinxton, Cambridge CB10 1SD, UK; European Molecular Biology Laboratory, European Bioinformatics Institute, Wellcome Genome Campus, Hinxton, Cambridge CB10 1SD, UK; European Molecular Biology Laboratory, European Bioinformatics Institute, Wellcome Genome Campus, Hinxton, Cambridge CB10 1SD, UK; European Molecular Biology Laboratory, European Bioinformatics Institute, Wellcome Genome Campus, Hinxton, Cambridge CB10 1SD, UK; European Molecular Biology Laboratory, European Bioinformatics Institute, Wellcome Genome Campus, Hinxton, Cambridge CB10 1SD, UK; European Molecular Biology Laboratory, European Bioinformatics Institute, Wellcome Genome Campus, Hinxton, Cambridge CB10 1SD, UK; European Molecular Biology Laboratory, European Bioinformatics Institute, Wellcome Genome Campus, Hinxton, Cambridge CB10 1SD, UK; European Molecular Biology Laboratory, European Bioinformatics Institute, Wellcome Genome Campus, Hinxton, Cambridge CB10 1SD, UK; European Molecular Biology Laboratory, European Bioinformatics Institute, Wellcome Genome Campus, Hinxton, Cambridge CB10 1SD, UK; European Molecular Biology Laboratory, European Bioinformatics Institute, Wellcome Genome Campus, Hinxton, Cambridge CB10 1SD, UK; European Molecular Biology Laboratory, European Bioinformatics Institute, Wellcome Genome Campus, Hinxton, Cambridge CB10 1SD, UK; European Molecular Biology Laboratory, European Bioinformatics Institute, Wellcome Genome Campus, Hinxton, Cambridge CB10 1SD, UK; European Molecular Biology Laboratory, European Bioinformatics Institute, Wellcome Genome Campus, Hinxton, Cambridge CB10 1SD, UK; European Molecular Biology Laboratory, European Bioinformatics Institute, Wellcome Genome Campus, Hinxton, Cambridge CB10 1SD, UK; European Molecular Biology Laboratory, European Bioinformatics Institute, Wellcome Genome Campus, Hinxton, Cambridge CB10 1SD, UK; European Molecular Biology Laboratory, European Bioinformatics Institute, Wellcome Genome Campus, Hinxton, Cambridge CB10 1SD, UK; European Molecular Biology Laboratory, European Bioinformatics Institute, Wellcome Genome Campus, Hinxton, Cambridge CB10 1SD, UK; European Molecular Biology Laboratory, European Bioinformatics Institute, Wellcome Genome Campus, Hinxton, Cambridge CB10 1SD, UK; European Molecular Biology Laboratory, European Bioinformatics Institute, Wellcome Genome Campus, Hinxton, Cambridge CB10 1SD, UK; European Molecular Biology Laboratory, European Bioinformatics Institute, Wellcome Genome Campus, Hinxton, Cambridge CB10 1SD, UK; European Molecular Biology Laboratory, European Bioinformatics Institute, Wellcome Genome Campus, Hinxton, Cambridge CB10 1SD, UK; European Molecular Biology Laboratory, European Bioinformatics Institute, Wellcome Genome Campus, Hinxton, Cambridge CB10 1SD, UK; European Molecular Biology Laboratory, European Bioinformatics Institute, Wellcome Genome Campus, Hinxton, Cambridge CB10 1SD, UK; European Molecular Biology Laboratory, European Bioinformatics Institute, Wellcome Genome Campus, Hinxton, Cambridge CB10 1SD, UK; European Molecular Biology Laboratory, European Bioinformatics Institute, Wellcome Genome Campus, Hinxton, Cambridge CB10 1SD, UK; European Molecular Biology Laboratory, European Bioinformatics Institute, Wellcome Genome Campus, Hinxton, Cambridge CB10 1SD, UK; European Molecular Biology Laboratory, European Bioinformatics Institute, Wellcome Genome Campus, Hinxton, Cambridge CB10 1SD, UK; European Molecular Biology Laboratory, European Bioinformatics Institute, Wellcome Genome Campus, Hinxton, Cambridge CB10 1SD, UK; European Molecular Biology Laboratory, European Bioinformatics Institute, Wellcome Genome Campus, Hinxton, Cambridge CB10 1SD, UK; European Molecular Biology Laboratory, European Bioinformatics Institute, Wellcome Genome Campus, Hinxton, Cambridge CB10 1SD, UK; European Molecular Biology Laboratory, European Bioinformatics Institute, Wellcome Genome Campus, Hinxton, Cambridge CB10 1SD, UK; European Molecular Biology Laboratory, European Bioinformatics Institute, Wellcome Genome Campus, Hinxton, Cambridge CB10 1SD, UK; European Molecular Biology Laboratory, European Bioinformatics Institute, Wellcome Genome Campus, Hinxton, Cambridge CB10 1SD, UK; European Molecular Biology Laboratory, European Bioinformatics Institute, Wellcome Genome Campus, Hinxton, Cambridge CB10 1SD, UK; European Molecular Biology Laboratory, European Bioinformatics Institute, Wellcome Genome Campus, Hinxton, Cambridge CB10 1SD, UK; European Molecular Biology Laboratory, European Bioinformatics Institute, Wellcome Genome Campus, Hinxton, Cambridge CB10 1SD, UK; European Molecular Biology Laboratory, European Bioinformatics Institute, Wellcome Genome Campus, Hinxton, Cambridge CB10 1SD, UK; European Molecular Biology Laboratory, European Bioinformatics Institute, Wellcome Genome Campus, Hinxton, Cambridge CB10 1SD, UK; European Molecular Biology Laboratory, European Bioinformatics Institute, Wellcome Genome Campus, Hinxton, Cambridge CB10 1SD, UK; European Molecular Biology Laboratory, European Bioinformatics Institute, Wellcome Genome Campus, Hinxton, Cambridge CB10 1SD, UK; European Molecular Biology Laboratory, European Bioinformatics Institute, Wellcome Genome Campus, Hinxton, Cambridge CB10 1SD, UK; European Molecular Biology Laboratory, European Bioinformatics Institute, Wellcome Genome Campus, Hinxton, Cambridge CB10 1SD, UK; European Molecular Biology Laboratory, European Bioinformatics Institute, Wellcome Genome Campus, Hinxton, Cambridge CB10 1SD, UK; European Molecular Biology Laboratory, European Bioinformatics Institute, Wellcome Genome Campus, Hinxton, Cambridge CB10 1SD, UK; European Molecular Biology Laboratory, European Bioinformatics Institute, Wellcome Genome Campus, Hinxton, Cambridge CB10 1SD, UK; European Molecular Biology Laboratory, European Bioinformatics Institute, Wellcome Genome Campus, Hinxton, Cambridge CB10 1SD, UK; European Molecular Biology Laboratory, European Bioinformatics Institute, Wellcome Genome Campus, Hinxton, Cambridge CB10 1SD, UK; European Molecular Biology Laboratory, European Bioinformatics Institute, Wellcome Genome Campus, Hinxton, Cambridge CB10 1SD, UK; European Molecular Biology Laboratory, European Bioinformatics Institute, Wellcome Genome Campus, Hinxton, Cambridge CB10 1SD, UK; European Molecular Biology Laboratory, European Bioinformatics Institute, Wellcome Genome Campus, Hinxton, Cambridge CB10 1SD, UK; European Molecular Biology Laboratory, European Bioinformatics Institute, Wellcome Genome Campus, Hinxton, Cambridge CB10 1SD, UK; European Molecular Biology Laboratory, European Bioinformatics Institute, Wellcome Genome Campus, Hinxton, Cambridge CB10 1SD, UK; European Molecular Biology Laboratory, European Bioinformatics Institute, Wellcome Genome Campus, Hinxton, Cambridge CB10 1SD, UK; European Molecular Biology Laboratory, European Bioinformatics Institute, Wellcome Genome Campus, Hinxton, Cambridge CB10 1SD, UK; European Molecular Biology Laboratory, European Bioinformatics Institute, Wellcome Genome Campus, Hinxton, Cambridge CB10 1SD, UK; European Molecular Biology Laboratory, European Bioinformatics Institute, Wellcome Genome Campus, Hinxton, Cambridge CB10 1SD, UK; European Molecular Biology Laboratory, European Bioinformatics Institute, Wellcome Genome Campus, Hinxton, Cambridge CB10 1SD, UK; European Molecular Biology Laboratory, European Bioinformatics Institute, Wellcome Genome Campus, Hinxton, Cambridge CB10 1SD, UK; European Molecular Biology Laboratory, European Bioinformatics Institute, Wellcome Genome Campus, Hinxton, Cambridge CB10 1SD, UK; European Molecular Biology Laboratory, European Bioinformatics Institute, Wellcome Genome Campus, Hinxton, Cambridge CB10 1SD, UK; European Molecular Biology Laboratory, European Bioinformatics Institute, Wellcome Genome Campus, Hinxton, Cambridge CB10 1SD, UK; European Molecular Biology Laboratory, European Bioinformatics Institute, Wellcome Genome Campus, Hinxton, Cambridge CB10 1SD, UK; European Molecular Biology Laboratory, European Bioinformatics Institute, Wellcome Genome Campus, Hinxton, Cambridge CB10 1SD, UK; European Molecular Biology Laboratory, European Bioinformatics Institute, Wellcome Genome Campus, Hinxton, Cambridge CB10 1SD, UK; European Molecular Biology Laboratory, European Bioinformatics Institute, Wellcome Genome Campus, Hinxton, Cambridge CB10 1SD, UK; European Molecular Biology Laboratory, European Bioinformatics Institute, Wellcome Genome Campus, Hinxton, Cambridge CB10 1SD, UK; European Molecular Biology Laboratory, European Bioinformatics Institute, Wellcome Genome Campus, Hinxton, Cambridge CB10 1SD, UK; European Molecular Biology Laboratory, European Bioinformatics Institute, Wellcome Genome Campus, Hinxton, Cambridge CB10 1SD, UK

## Abstract

The Ensembl project (https://www.ensembl.org) annotates genomes and disseminates genomic data for vertebrate species. We create detailed and comprehensive annotation of gene structures, regulatory elements and variants, and enable comparative genomics by inferring the evolutionary history of genes and genomes. Our integrated genomic data are made available in a variety of ways, including genome browsers, search interfaces, specialist tools such as the Ensembl Variant Effect Predictor, download files and programmatic interfaces. Here, we present recent Ensembl developments including two new website portals. Ensembl Rapid Release (http://rapid.ensembl.org) is designed to provide core tools and services for genomes as soon as possible and has been deployed to support large biodiversity sequencing projects. Our SARS-CoV-2 genome browser (https://covid-19.ensembl.org) integrates our own annotation with publicly available genomic data from numerous sources to facilitate the use of genomics in the international scientific response to the COVID-19 pandemic. We also report on other updates to our annotation resources, tools and services. All Ensembl data and software are freely available without restriction.

## INTRODUCTION

Ensembl accelerates worldwide genomic research by integrating, harmonizing and annotating genome data and disseminating it via a coherent and consistent set of interfaces and tools. We import primary data from archive resources such as INSDC (1), dbSNP (2) and the European Variation Archive (EVA, https://www.ebi.ac.uk/eva), and add value via detailed and comprehensive annotation of transcript structures (3), genomic variants (4) and regulatory regions (5). We also enable the study of evolution by large-scale comparison of genomes and gene products across many species (6). These data can be accessed via our website, programmatically via a number of application programming interfaces (APIs) (7,8), and downloaded in numerous standard file formats. We develop and make available a variety of tools for genomic analysis, including the Ensembl Variant Effect Predictor (VEP) (9). Our software, database and tools infrastructure is freely available and is used to power the nonvertebrate genome resources provided by the clade-specific Ensembl Genomes websites (10), collaborating resources such as WormBase (11), and community-oriented databases focused on branches of the taxonomy, such as AvianBase (12) and LepBase (http://lepbase.org).

Genome research has evolved significantly since the publication of the human genome 20 years ago (13). Genome medicine is developing rapidly across the world as a diagnostic tool for rare diseases, for prioritization and selection of cancer treatments, and for many other applications. At the same time, we are in the midst of the sixth mass extinction (14), and loss of biodiversity is entwined with many of the key challenges faced by human society today including pandemic zoonotic diseases, climate change, food security, availability of drugs and vaccines, and renewable energy. Genomics will play an increasingly important role in biomedical and biodiversity science, and a key aim for Ensembl is to evolve accordingly by developing our reference annotation resources and genomics infrastructure platform to support these applications.

This year, we have enhanced our reference transcript, regulatory and variation annotation in human, vertebrate model organisms, and other species of high socioeconomic importance. For example, our human genome resources have seen three updates to the Ensembl/GENCODE reference transcript set, continued collaborative work within the Matched Annotation from NCBI and EMBL-EBI (MANE) project, enriched regulatory data for enhancer activity and microRNA-gene interactions, and variation annotation incorporating new structural variants and allele frequency data.

Large-scale biodiversity sequencing projects, such as the Vertebrate Genomes Project (https://vertebrategenomesproject.org/) and the Darwin Tree of Life Project (https://www.darwintreeoflife.org/), are generating high-quality genome assemblies at an accelerating pace. Supporting these projects and others under the umbrella of the Earth BioGenome Project (15) is a priority development area for us. We previously reported on our efforts to re-engineer our evidence-based automated transcript annotation pipeline to achieve an order-of-magnitude increase in efficiency (16). This year, we have consolidated this work to deliver our largest annual increase in supported genomes, while creating a new mechanism—Ensembl Rapid Release—for the dissemination and distribution of genomes as soon as they are annotated. We have also created a sub-portal of Ensembl for the Vertebrate Genomes Project, which acts as a template for further project or community-specific windows into Ensembl data.

As well as an annotation resource, Ensembl is also a comprehensive technology platform for the management, analysis and dissemination of genomic data. We have enriched our website and services with new ways of accessing data, and have developed a new interface for exploring multidimensional Track Hubs. At the same time, work on our redesigned website (17) and APIs continues apace, with recent innovations including a new prototype API.

The COVID-19 pandemic is unprecedented in its mobilization of the global scientific community, with many researchers refocusing their work to help understand the disease, treat its symptoms and slow its transmission. We have contributed to the effort by creating a browser and tools for the genome of the severe acute respiratory syndrome coronavirus 2 (SARS-CoV-2) virus that causes COVID-19. The reference sequence acts as a foundation for exploring data pertaining to the molecular genetics of virus transmission and pathogenicity, and our established tools such as the Ensembl VEP are a useful platform for exploring the variation between viral isolates.

## REFERENCE ANNOTATION FOR KEY SPECIES

We have enriched our reference human transcript annotation, which is created as part of the GENCODE consortium (18). One area of focus this year has been re-examining and extending the annotation for genes implicated with COVID-19 infection and response, which has resulted thus far in the addition or amendment of over 4000 transcripts. These data are currently available as a Track Hub (19), and will be made available as part of the full Ensembl/GENCODE transcript set in an imminent Ensembl release. We previously reported (17) on a new collaboration with the NCBI—MANE Select—that aims to produce a single reference transcript for every human protein gene with identical structure between Ensembl/GENCODE and RefSeq (20). In our recent v0.91 of MANE Select (July 2020), 84% of human protein-coding genes are covered (up from 67% last year). We have been working closely with clinical partners to prioritize loci for inclusion in MANE Select, and we expect close to full coverage of all clinical genes with confirmed association with human disease to be available imminently. This includes the 59 genes considered to be clinically actionable by the American College of Medical Genetics and Genomics (ACMG) (21).

We have broadened and deepened our reference human regulatory annotation. This year, we incorporated updated enhancer activity data from VISTA (22) and miRNA/gene interactions from Tarbase v8 (23). We also refreshed our regulatory annotation for GRCh37, in recognition of the widespread use of this previous human assembly. Our GRCh37 regulatory annotation now represents over 100 human epigenomes, cataloguing over half a million elements covering 16% of the genome from over 9TB of experimental data from the Roadmap Epigenomics (24), ENCODE (25) and BLUEPRINT (26) projects.

Our high-quality reference human gene and regulatory annotation underpin the interpretation of genetic variation in human populations. We have enhanced our variant collections by updating to the latest versions of multiple resources including dbSNP and the Genome Aggregation Database (gnomAD, (27)), and added new human allele frequency data from the Gambian Genome Variation project (28). We have also integrated new structural variant data from sources including gnomAD, ClinVar (29) and the NCBI Curated Common Structural Variants set (https://www.ncbi.nlm.nih.gov/dbvar/studies/nstd186).

Beyond human, provision of annotation resources for model organisms and species of socioeconomic importance continues to be a key activity area. For example, we are part of the AQUA-FAANG consortium (https://www.aqua-faang.eu), which is focused the functional genomics of the six most economically important fish species in European aquaculture: European Seabass, Gilthead Seabream, Atlantic Salmon, Rainbow Trout, Common Carp and Turbot. We have produced detailed gene annotations for all six species, with work underway to add regulatory annotation. We have also integrated variant data for Atlantic Salmon from the EVA.

We support multiple strain/breed genomes for model organisms and farmed/companion animals, and we previously reported on the incorporation of 17 laboratory mouse strains (30) and 12 pig breeds (17). This year we have added additional breed genomes for sheep (Rambouillet), goat (Black bengal) and dog (Great Dane, Banshi and German Shepherd) as well as three strains of common carp.

## SUPPORTING BIODIVERSITY GENOMICS

**Figure 1.**
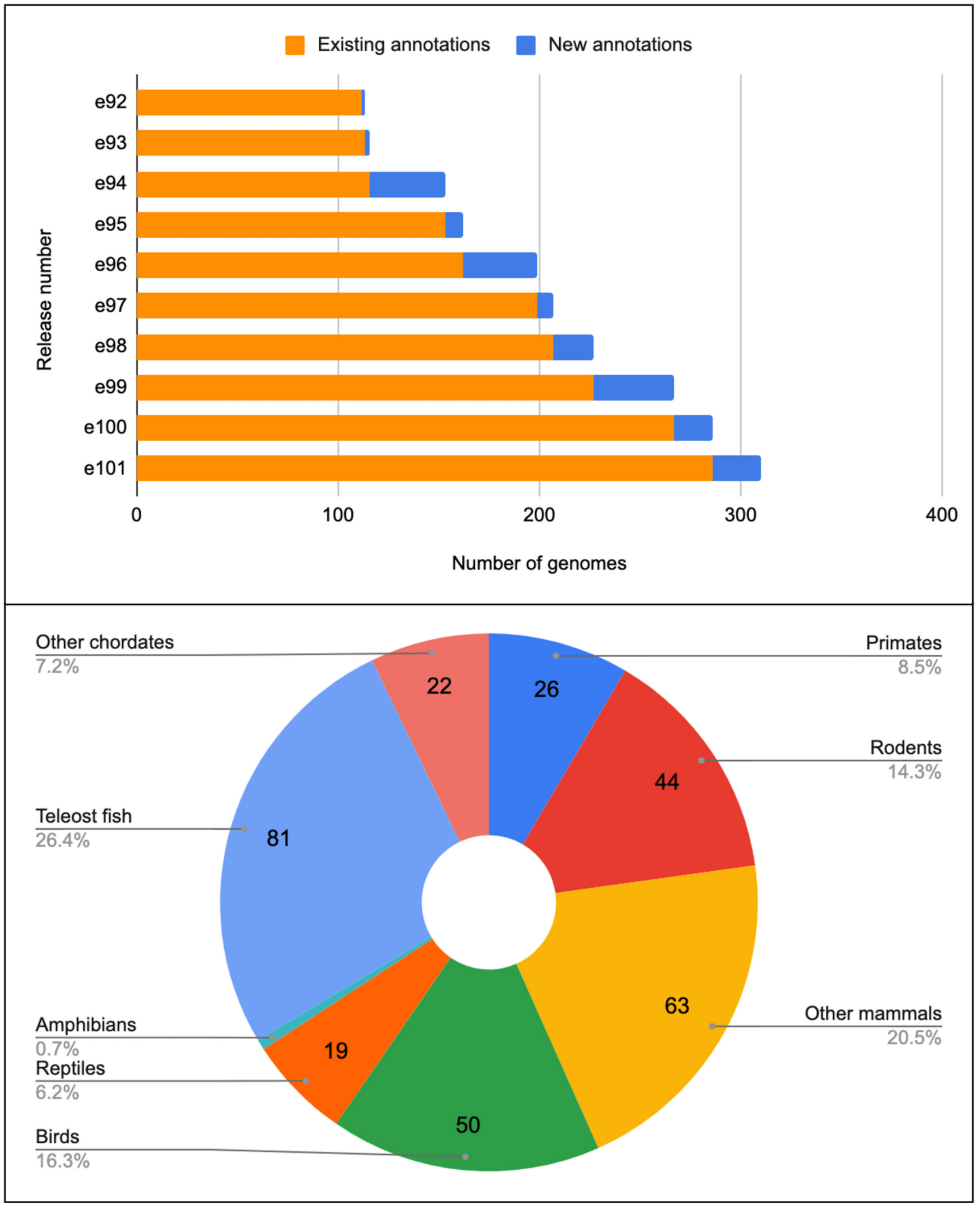
Growth and representation of genomes in Ensembl. Top panel: Ensembl genome counts for the 10 most recent Ensembl releases. Since Ensembl release 92 (April 2018), we have nearly tripled the number of genomes we support. Lower panel: distribution of current chordate genomes in Ensembl, by clade.

In the past year, we have annotated and integrated 83 new vertebrate genomes across diverse clades (see Figure 1). Notable among the additions are kakapo, golden eagle, small tree finch, wild duck, Indian cobra, mainland tiger snake and tuatara (31).

Our plans to rapidly increase the number of genomes that we annotate, compare and disseminate require us to make continual improvements to our gene annotation and comparative genomics workflows. We are focused both on increasing throughput and most effectively using the available experimental evidence to increase annotation accuracy. This year, we have enhanced our gene annotation pipeline with a new module for incorporating long-read transcriptomic data produced by the Oxford Nanopore Technologies platform. This was first used for annotating the kakapo genome and allows us to more confidently capture full-length transcript structures including a better representation of untranslated regions.

The traditional Ensembl data distribution model is one of periodic integrated and versioned releases in which every genome is comprehensively annotated, compared to other genomes and made available via our complete set of tools and services. Integrated releases involve the complex orchestration of a number of large-scale analyses and processes across the whole Ensembl project. This means that there can sometimes be a delay of multiple months between our production of the primary gene and transcript annotation and when a genome is available via an Ensembl release.

The redesigned Ensembl genome browser (see below) will support the release of genomes quickly after annotation and allow for their exploration via a core set of functionality and tools before their incorporation into an integrated release. In advance of the release of our new website and in support of the Darwin Tree of Life and other emerging nodes of the Earth BioGenome Project, we have created a new sub-portal of our current website for early access to annotated genomes. Ensembl Rapid Release (https://rapid.ensembl.org) is updated every 2 weeks with new genomes being added quickly after primary annotation. Core functionality on the site includes (i) a genome browser for every genome with tracks for primary gene annotation and repeats; (ii) functional annotation of annotated gene products using InterProScan (32); (iii) BLAST search of user-supplied sequences against the genome and its gene sequences; and (iv) download files of the genome and annotations in a variety of standard formats. Ensembl Rapid Release hosts both vertebrate and nonvertebrate genomes with the latter to be integrated into the appropriate Ensembl Genomes website (10). In the next year, we plan to add additional functionality to Ensembl Rapid Release, including homologies, gene symbols, additional genome browser tracks and programmatic access.

As we add more genomes to Ensembl, finding relevant data collections becomes increasingly important. To this end, we are creating community-oriented gateways for specific collections of genomes based initially on nodes of the Earth BioGenome Project. These act as landing pages for the annotated genomes arising from these projects with easy one-click access to key resources such as annotation files and the genome browser. Our first such gateway is for the Vertebrate Genomes Project (https://projects.ensembl.org/vgp) and one for the Darwin Tree of Life is planned for the next year. In future, we will extend the concept to create genome collections and sub-portals aimed at specific scientific communities such as agricultural genomics or parasitology and pathology.

## TOOLS AND SERVICES

The capabilities of the Ensembl VEP have been expanded in several ways over the past year. For example, the Ensembl VEP can now standardize the representation of ambiguous insertion/deletion variants prior to consequence prediction, which addresses anomalous differences in predicted molecular consequences when variants in repetitive genomic regions are described in multiple equivalent ways. Another major enhancement is the reporting of variant synonyms, which enables improved navigation to additional information in resources such as ClinVar, UniProt and PharmGKB (33). Other improvements include enriched annotation of potential splicing impact (using scores from SpliceAI (34)), incorporation of phenotype association and variant citation data from DisGeNET (35), and protein annotations from neXtProt (36).

We previously introduced the Ensembl Transcript Archive (Tark) (17), which tracks changes to transcript structures across different annotation sources, releases and genome builds (http://dev-tark.ensembl.org). Ensembl Tark now includes all versioned human transcript sets from RefSeq and Ensembl/GENCODE, and contains an up-to-date list of MANE Select transcripts. In the next year, we will add support for Locus Reference Genomic (37) reference sequences.

**Figure 2.**
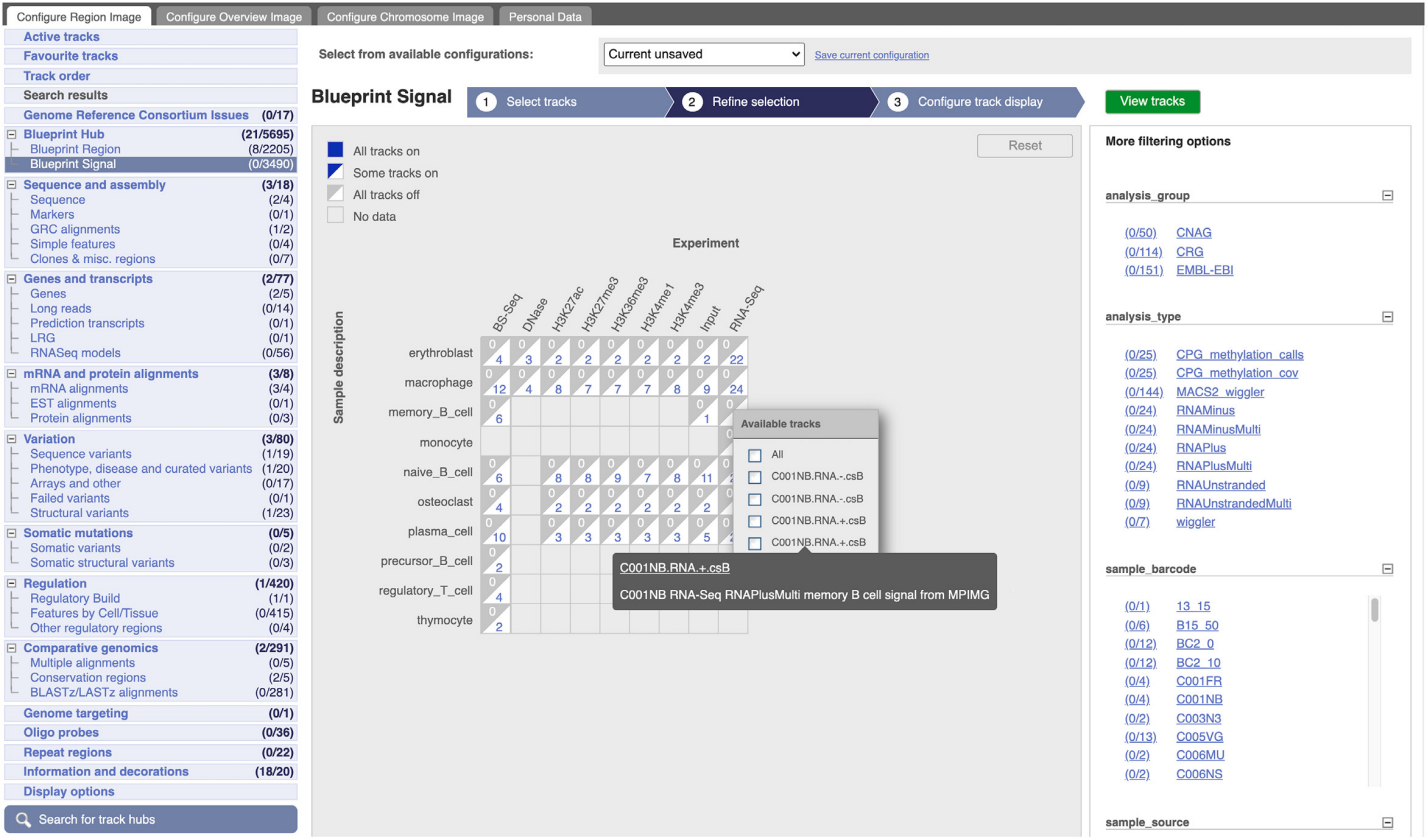
The Blueprint Signal Track Hub. The primary dimensions of the Hub, Sample description and Experiment, are used to create the track selection matrix. Other dimensions within the data are shown in a panel on the right (e.g. Analysis group and Analysis Type), and tracks can be filtered by clicking on any of the types within those. Even greater granularity of track selection can be achieved by selecting tracks from a pop-up menu associated with each individual cell of the matrix.

We have made numerous improvements to our main website portal (https://www.ensembl.org). An example is our redesigned interface for configuring multi-dimensional Track Hubs. For 2D Hubs, we present a simple matrix as before, but where the Track Hub provider uses more than two dimensions the new interface allows for easier discovery and selection of these tracks (see Figure 2).

Our new genome browser is currently in beta at http://2020.ensembl.org. We have recently focused on the development of key workflows such as viewing summary information about a gene, selecting transcripts and downloading sequence information. In tandem, we are developing a new API designed for the needs of modern, responsive web applications based on the GraphQL standard (graphql.org). The Ensembl GraphQL API enables the fine-grained request and delivery of specific slices of data. It is initially being developed primarily for our new website, and we aim for a public prototype in the next year.

## SUPPORTING COVID-19 GENOMICS

COVID-19 is caused by SARS-CoV-2, which has spread across the globe since emerging in late 2019. Our SARS-CoV-2 genome browser and related resources (https://covid-19.ensembl.org) support genomics-based approaches to the study of the virus.

The reference sequence represented in Ensembl (INSDC accession GCA_009858895.3) is the RNA genome isolated from one of the first cases in Wuhan (38) and is widely used as a standard reference for variant calling and to root phylogenetic analyses. We have annotated the reference genome with genic features using a slightly adapted version of our annotation pipeline. Annotation of the ORF1ab/ORF1a locus in particular is complicated by a programmed-1 ribosome slippage in the translation of one of its two polyprotein products (39). The Ensembl data-model and database schema allows us to represent this situation elegantly as a sequence edit, allowing our annotation pipeline to store the transcript structure correctly and our API to produce the correct peptide products.

To enrich our gene structure annotation, we have added structural RNAs from Rfam (40), full cross-references to annotated entries at Uniprot (41) and RefSeq, functional annotation from the Gene Ontology Consortium (42), and annotation of protein domains from InterProScan. For the latter, we created a genome browser track projecting the protein-domain annotations onto the genome, facilitating a genome-oriented view of the gene projects (including the nonstructural cleavage products of ORF1a/ORF1ab). We also display a collection of other tracks on the genome browser, including the annotation that was submitted to INSDC as part of the publication of the reference genome (38), and community annotation of sites and regions resulting from an effort coordinated by the UCSC genome browser (43).

We are integrating and annotating identified variants in the genome of SARS-Cov-2 using Ensembl VEP. This has been an active area of research during the pandemic, with many countries undergoing concerted programs of isolate sequencing. For example, the COVID-19 data portal (https://www.covid19dataportal.org) has developed a pipeline employing LoFreq (44) to identify variants in publicly available SARS-CoV-2 genomes sequenced and deposited in the European Nucleotide Archive. We currently display variants from 5106 SARS-CoV2 samples, with significant growth expected in future releases. To complement our own analysis of the public data, we also display annotated variants from real-time pathogen surveillance resource Nextstrain (45), using sequence data acquired from the virus data sharing platform GISAID (46). As reported by De Maio *et al.* (https://virological.org/t/issues-with-sars-cov-2-sequencing-data/473), some sites in the SARS-CoV-2 are associated with unreliable variant calls, due to artifacts in sample preparation, sequencing technology and consensus calling. We have created a genome browser track for these sites to display which variants identified in the various datasets should be treated with caution.

Future releases of the portal will include new data as it emerges, additional tools (including sequence search) and comparative genomics views to enable the comparison of SARS-CoV-2 genome and gene products with those of related coronaviruses found in other species.

## USER SUPPORT AND TRAINING

We maintain a close relationship with our global user community via our helpdesk (https://www.ensembl.org/Help/Contact or helpdesk@ensembl.org), and through our developer mailing list (https://lists.ensembl.org/mailman/listinfo/dev) we facilitate a network of bioinformaticians using the Ensembl platform.

We also directly train researchers throughout the world in the use of the Ensembl (http://training.ensembl.org). Traditionally, we have designed in-person courses aimed at wet-lab researchers and clinicians, developers and educators, with all courses tailored to suit the needs of a host institute or to fit in as part of a series. The COVID-19 pandemic has seen nearly all of our training in 2020 delivered on-line. This included an Ensembl component in a virtual training course for the Pan African Bioinformatics Network for the Human Heredity and Health in Africa consortium, H3ABioNet, reaching >1300 participants across 16 countries. We remain committed to in-person training but acknowledge that virtual training will occupy a larger proportion of our portfolio in the post-COVID-19 world.

## CONCLUSION

While Ensembl resources are used in many different ways by thousands of researches across the world, our mission can be viewed from three broad perspectives. The first is enabling the fine-grained interpretation of genomic variation via the provision of comprehensive reference annotation for human, model organisms and other species of socioeconomic importance exemplified by our updates of the MANE Select transcript set and our enhanced reference regulatory and variation annotation resources. The second perspective is enabling genomic approaches to the study of biodiversity, this year seeing improvements to the breadth and depth of genomes we annotate and serve and the development of the Ensembl Rapid Release platform. The third perspective is enabling genome bioinformatics via the development of an extensive and reusable genomics infrastructure platform, with improved Ensembl Tark, enhanced website configuration options and accelerated work on our new genome browser. Finally, cutting across these three perspectives, we have integrated Ensembl's first viral genome to create our SARS-CoV-2 genome browser.

## DATA AVAILABILITY

All Ensembl integrated data are available without restriction from our website (https://www.ensembl.org), in bulk from our FTP site (ftp://ftp.ensembl.org) and programmatically via our REST API (https://rest.ensembl.org). Ensembl code is available from GitHub (https://github.com/Ensembl) under an open source Apache 2.0 license. News about our releases and services can be found our blog (https://www.ensembl.info), our announce mailing list (https://lists.ensembl.org/mailman/listinfo/announce), Twitter (@ensembl) and Facebook (https://facebook.com/Ensembl.org).
